# On the Non-identity Causal Theory of Spacetime from Causal Set Theory

**DOI:** 10.1007/s10670-024-00836-1

**Published:** 2024-08-29

**Authors:** Rasmus Jaksland, Niels Linnemann

**Affiliations:** 1 Department of Science Education, University of Copenhagen, Copenhagen, Denmark; 2 Department of Philosophy, University of Geneva, Geneva, Switzerland

## Abstract

The aim to provide a causal theory of spacetime is not new. The overall program, however, was largely deemed unsuccessful, chiefly due to criticism voiced by Smart (Monist 53:385–395, 1969), Nerlich (Br J Philos Sci 33(4):361–388, 1982) and Earman (Synthese 24:74–86, 1972). Recently, Baron and Le Bihan (Noûs 58:202–224, 2023) have argued that developments in contemporary physics should make us reconsider this verdict. More precisely, they argue the emergence of spacetime from causal set theory (CST), where “the metric structure of spacetime can be recovered from its causal structure” (Baron and Le Bihan 2023, 2), “suggests a very natural way to reformulate the causal theory of spacetime” (ibid., 9)—an account which they end up dubbing the non-identity causal theory. This paper questions the success of Baron and Le Bihan’s non-identity theory. It is shown that (1) the specific grounding Baron and Le Bihan propose for timelike and spacelike relations is not plausible even when charitably reconstructed; and (2) that a causal theory of spacetime based on general relativity is just as successful for establishing a non-identity theory as a theory based on CST. In short then, we argue that the causal theory of spacetime proposed by Baron and Le Bihan is supported just as well (or badly) by the physics that already took centre stage in the original discussions of the causal theory of spacetime.

## Introduction

The aim to provide a causal theory of spacetime is not new (Grünbaum, 1973; Reichenbach, 1956; Van Fraassen, 1970). The overall program, however, was largely deemed unsuccessful, chiefly due to criticism voiced by Smart (1969), Nerlich (1982) and Earman (1972). Recently, Baron and Le Bihan (2023) have argued that developments in contemporary physics should make us reconsider this verdict. More precisely, they argue that the way spacetime emerges from causal set theory (CST), where “the metric structure of spacetime can be recovered from its causal structure” (Baron & Le Bihan, 2023, 2), “suggests a very natural way to reformulate the causal theory of spacetime” (Baron & Le Bihan, 2023, 9)—an account they call the non-identity causal theory.

Traditionally, proponents of the causal theory of spacetime have argued that spacetime relations are identical to causal relations. In the context of CST such an identity theory still fails, or so Baron and Le Bihan argue. However, they make the case that a non-identity version succeeds in the CST context. Their non-identity theory is characterised by the claim that “spatiotemporal relations are grounded in more fundamental causal relations between events” (Baron & Le Bihan, 2023, 15). Here, grounding is a placeholder for a generic ontological dependence relation which is consistent with spatiotemporal relations being distinct from causal relations. With this change from identity to grounding, they insist that this non-identity theory “finds support from contemporary physics” (Baron & Le Bihan, 2023, 21) in the form of CST.

This paper questions the success of Baron and Le Bihan’s theory. In the first part (Sects. 2 and 3), it is shown that the specific grounding Baron and Le Bihan propose for timelike and spacelike relations is not plausible even when charitably reconstructed. In the second part (Sect. 4), it is shown that a causal theory of spacetime based on General Relativity (GR) is just as successful for establishing a non-identity theory as one based on CST. We argue that the causal theory of spacetime proposed by Baron and Le Bihan is supported just as well (or badly)^1^ by the physics that already took centre stage in the original discussions of the causal theory of spacetime.

## The Non-identity Theory from Causal Sets

Baron and Le Bihan’s non-identity causal theory of spacetime seeks to *ground* spatiotemporal relations in causal relations. That such grounding is indeed possible, Baron and Le Bihan argue, is suggested by CST. This section, however, makes clear that the support from CST comes at the price of adopting a view of spacetime and causation compatible with CST.

After introducing CST and Baron and Le Bihan’s associated non-identity theory (Sect. 2.1), we specifically categorize the notion of causation adhered to by Baron and Le Bihan in their proposal (Sect. 2.2), and clarify what spacetimes CST can at most ground (Sect. 2.3).

### Causal Set Theory and the Non-identity Theory

Causal set theory is an approach to quantum gravity that proposes discrete partially ordered sets of events (with a local finiteness condition between any two related events^2^ for the amount of sets in between them) as the higher-energy substructures from which classical general relativistic spacetimes can be recovered. More precisely, a causal set is a set *C*, which we shall refer to as a ‘causet’, with a partial order $\prec$ that satisfies the following conditions:

*Reflexive*: For all $x \in C$, we have $x \prec_C x$.
*Antisymmetric*: For all $x, y \in C$, we have $x \prec_C y$ and $y \prec_C x$ implies $x = y$.
*Transitive*: For all $x, y, z \in C$, we have $x \prec_C y$ and $y \prec_C z$ implies $x \prec_C z$.
*Locally finite poset*: For all $x, z \in C$, we have $\{y \in C | x \prec_C y \prec_C z\}$ is a finite set.

A large sector of general relativistic spacetimes are conjectured to be recoverable from discrete causets: just as the continuum fluid is an approximation to the exact molecular configuration making up the fluid, continuum spacetimes are approximations of the underlying causal set structures; and in particular, just as the same continuum fluid is approximated by many different molecular configurations, the same continuum spacetime will be approximated by many different causets.^3^

The causal set approach to spacetime is motivated by the result from GR that, for certain spacetimes, the continuous metric can be obtained from causal structure and local volume information. ‘Causal structure’ here denotes the structure that determines which events are connectible via a causal path, i.e., a path that consists of segments of null and/or timelike curves; the causal structure here is captured by $(M, \prec _M)$ where for $x, y \in M: x \prec _M y$ if there exists a future-directed causal (non-spacelike) path from *x* to *y*. Together with local volume information, which is about how many events are there in a manifold region, $(M, \prec _M)$ determines the metric provided that the spacetime is future- and past-distinguishing^4^ (see Malament, 1977), which is, just to be clear, far from being the case for all GR models. Within the discrete structure of CST, the (discrete) causal structure is explicitly captured by the causet and the local volume information is captured by the number of discrete events within a region. Upon taking the continuum limit for a discrete causal structure (zooming out, if you want), these should together uniquely determine a classical spacetime metric. 5

One aspect of how spacetime is approximated by a causet is central for what follows, so let us describe more precisely how causets approximate spacetimes.^6^ One necessary condition for a notion of a causet *C* approximating a continuum spacetime (*M*, *g*) is that there is a specific many-to-one embedding of the causet into that spacetime that preserves the causal structure $\prec _C$ when embedded into (*M*, *g*):

An *embedding* [of a causet *C*] is the injective map

$$\begin{aligned} \Phi : C \hookrightarrow (M, g), x \prec _C y \Leftrightarrow \Phi (x) \prec _M \Phi (y), \end{aligned}$$

where $\prec _C$ and $\prec _M$ denote the causal order relations in *C* and *M* respectively (Surya, 2019, 14)

In other words, if an event succeeds another in the causet, then the corresponding embedded event must also succeed the other corresponding embedded event in (*M*, *g*); the causal structure of the causet must agree with that of the spacetime it approximates.

Even if a causal set might fit onto a spacetime with respect to causal structure, it is in general not the case that counting the number of events in the causal structure mapped onto a region of (*M*, *g*) adequately reproduces the volume size of that region. Therefore one in fact requires something slightly stronger, namely what is called a ‘faithful’ embedding, for a satisfactory notion of spacetime approximation through a causal set:

A causal set [*C*] is said to approximate a spacetime $C \sim (M, g)$ at density $\rho _C = V_c^{-1}$ if there exists a *faithful embedding* [, i.e. an embedding] ${\Phi : C \hookrightarrow M}$ [, $x \prec _C y \Leftrightarrow \Phi (x) \prec _M \Phi (y)$, where] $\Phi (C)$ is a uniform distribution in (*M*, *g*) at density $\rho _C$ (Surya, 2019, 14)

Here, ‘uniform’ is relative to the volume measure on (*M*, *g*). In particular, the notion of faithful embedding—and that of ‘uniform distribution’ specifically—allows for capturing that (*) a certain number of (somewhat neighbouring) causal set elements *n* stands in correspondence to a spacetime volume *V*, i.e. $n = \rho _C V$ for fixed density $\rho _C$.

The required uniform distribution on the spacetime manifold is standardly explicated by the CST community as only realized effectively: it results from a Poisson distribution^7^ according to which the probability of finding *n* elements in a volume *v* of the corresponding spacetime is $P_v(n) = \frac{(\rho _c v)^n}{n!} \exp ({-\rho _c v})$; then, on average, i.e., at a sufficient level of coarse-graining, the number of events in the region with volume *v* is $\langle n \rangle = \rho _c v$; it is in this sense then that (*) is realised. With the concrete distribution underlying the requirement of ‘uniform distribution’ in hand, one can formulate a precise notion of approximation of a spacetime through a causal set (rather than vice versa): The spacetime (*M*, *g*) is said to (continuously) approximate a causal set *C* if *C* is an element of an ensemble of causal sets, $C \in {\mathcal {C}}(M, \rho _C)$ that arises from the following procedure: (1) randomly sprinkle ‘elements’ in accordance with the Poisson distribution onto (*M*, *g*); (2) connect these ‘elements’ in accordance with the causal structure of (*M*, *g*) to obtain a causal set; (3) as the process is random, a whole (countably infinite) ensemble of causets, ${\mathcal {C}}(M, \rho _C)$, is associated with it.

Now, that the definition of how a causal set approximates a spacetime (and its converse formulation) is apt is supposed to be corroborated by the expectation of the technical result that the notion of spacetime approximation through a causal set is exact down to the ‘cut off’ density scale $\rho _C$, i.e., “*C* can be faithfully embedded at density $\rho _C$ into two distinct spacetimes, (*M*, *g*) and $(M', g')$ iff they are approximately isometric” (Surya, 2019, 19). The status of this important result (known as *Hauptvermutung*) is, however, still only one of a conjecture.

With this explication of causal theory at hand, we can follow Baron and Le Bihan in their formulation of the non-identity causal theory of spacetime as follows:

Spatiotemporal relations are grounded in the presence or absence of fundamental causal connections between events that are embedded in a total causal structure ${\mathcal {C}}$ [*C* in our notation] where (1) ${\mathcal {C}}$ is governed by laws that qualify it to be a grounding base for spacetime and (2) the laws on ${\mathcal {C}}$ determine the modal status of each presence or absence of a causal connection within that structure (Baron & Le Bihan, 2023, 14).

Explicating this theory will be an ongoing task of this paper, and we will therefore return to this quote at several junctions. For now, what is important to notice is the role of CST in the non-identity causal theory of spacetime. Condition (1) specifies that the grounding base, the ‘causal’-part of the ‘causal theory of spacetime,’ is a CST causet and one that recovers a spacetime, presumably the spacetime that the spatiotemporal relations in question belong to. The development of CST in physics, in other words, appears to be crucial for this proposal and therefore for the new progress on the causal theory of spacetime. In relying on CST like this, however, Baron and Le Bihan inevitably come to depend on its understanding of the notions of ‘causal’ and ‘spacetime’.

### Causation

While Baron and Le Bihan, in general accordance with a causal theory of spacetime, state that spatiotemporal relations are grounded in fundamental causal connections, the causal connections in question are those that obtain between events of a causet in virtue of its partial order. The causal nature of these connections can too easily be taken for granted because of their origin in models of a theory known as *causal* set theory. At the outset, however, this is just a name given to an approach where models of GR are approximated by partially ordered discrete sets of events. That the theory is called *causal* set theory has to do with the GR structure that the causets manifestly capture, namely the causal structure $(M, \prec _M)$ introduced earlier.

The general relativistic causal structure is called ‘causal’ because it specifies between what events in spacetime a signal can be sent using a massive or massless point probe which are idealisable as moving along causal paths, i.e., they capture the possibility for one event to influence the other assuming that no influence can travel faster than the speed of light.^8^ As mentioned above, the metric structure of distinguishing GR spacetimes can be determined by exactly this general relativistic causal structure and the local volume element (also known as the local conformal factor). The general relativistic causal structure can also be represented by a conformal equivalence class of Lorentzian metrics (conformal structure)—or, more pictorially speaking, by a field of local light cones across the manifold of events. Now, a discretized version of this structure is precisely what a causet manifestly models. Indeed, the central requirement, introduced above, for how the causet approximates a GR model is that the embedding must preserve causal structure, i.e., for any two elements that are partially ordered in the causet (i.e., obey the relation $\prec_C$), their embedded counterparts are partially ordered on the spacetime (i.e., obey the relation $\prec_M$).

While a causet that is embedded into a manifold like this can thereby inherit the status as a causal structure in the GR sense, not all connections through the causet qualify as “causal connections” in the sense of Baron and Le Bihan. For convenience, let us refer to connections involving more than two events in the causet as *chains* to distinguish them from the connections via *paths* on the GR manifold. Not all chains in the causet are considered causal by Baron and Le Bihan because not all chains of events are such that a signal can travel through all the events on a causal path in the corresponding GR spacetime. Indeed, this will only be so if the chain between two events observes the partial order relation $\prec_C$, i.e., if we can move from one event to the other through the causet by only going from proceeding to succeeding events. Only chains that observe the partial order relation—what we shall denote *causal chains*—qualify as the “fundamental causal connections between events” that can be used to ground spatiotemporal relations in Baron and Le Bihan’s non-identity causal theory of spacetime. (The same is of course well-familiar for paths: not all paths are causal paths.)

Baron and Le Bihan’s understanding of ‘causal’ is, in this way, decisively that of CST and, therefore, informed by the use of this notion in GR—and *not*, in the first instance, by how causality is theorized in metaphysics. They do suggest, though, that an interventionist account of causation might agree with the notion of causation that CST inherits from GR (Baron & Le Bihan, 2023, sec. 5.2). We are happy to grant Baron and Le Bihan all of this. For present purposes, what is important is just to subsequently keep in mind that ‘causal’ and ‘causation,’ to connect with CST that forms the basis of the non-identity theory, must be understood in the sense originating in GR.

### Spacetime

In the statement of their non-identity causal theory of spacetime, Baron and Le Bihan include the condition (1) that “${\mathcal {C}}$ [*C* in our notation] is governed by laws that qualify it to be a grounding base for spacetime”. Since different causets will, in general, approximate different spacetimes (if they approximate a spacetime at all), a relevant qualification to add is that the spacetime approximated by ${\mathcal {C}}$ should be the one whose spatiotemporal relations one seeks to ground. This may seem rather obvious, but it makes a condition explicit: that the non-identity theory can, at best, ground spatiotemporal relations that belong to a spacetime that can be recovered by a causet. What these spacetimes are will be the topic below.

Baron and Le Bihan themselves notice that *no* GR spacetime can actually be completely recovered by a causet because general relativistic models have a continuum of points while a causet is discrete (so, even if infinite, only countably so). “Rather”, as they explain, “what we aim to do is ground spacetime points up to a certain scale factor, where that scale factor is a proposed discreteness cut-off” (Baron & Le Bihan, 2023, 12). Indeed, what CST intends to recover—with the Hauptvermutung on this point still only being a conjecture—is only an *approximation* to a GR spacetime. This is of course not surprising: CST is a different theory than GR; causal set theory is conjectured to reduce a sector of GR; and whenever (or at least usually when) one theory reduces a subsector of another theory, the models of the reducing theory will strictly speaking only approximate the models of the reduced theory.

One might argue that if one really wanted a causal theory of a relativistic spacetime down to an arbitrarily small length scale, then the grounding proposal based on the reducing theory of CST could not deliver it. This, however, would be to construe the problem too narrowly: Compare this to the example that Baron and Le Bihan (2023, 9) uses for other purposes that water is grounded in discrete $\mathrm {H_2 O}$ molecules. This grounding claim is not challenged even though we might sometimes treat water as a continuous substance and might in the past have thought that water was continuous. Likewise, even if we typically assume that relativistic spacetimes are continuous, this should, in analogy, not rule out that we might discover that the relativistic spacetime of the actual world is grounded in a discrete structure.

In fact, one might say that one wants a causal theory of a structure that represents spacetime while also representing its breakdown—and such a notion of structure could exactly be given by the notion of approximately equivalent metric relevant for the Hauptvermutung. However, in contrast with the discreteness of water, it arguably remains undecided whether spacetime is discrete, though there is (theoretical) evidence that it might be (see, e.g., Hossenfelder, 2013; Huggett & Wüthrich, 2013). Thus, whether one can even hope to use CST for a causal theory of spacetime for the actual world depends on this so far undecided empirical question.

Another important caveat already alluded to above is that the spacetimes that can be approximately recovered by CST form, at best, only a subset of GR spacetimes: there are spacetimes and, therefore, spatiotemporal relations that will, by all that we know today about CST, not be approximable in CST. Most immediately, the condition that the causet is antisymmetric—if $x {\prec_C} y$ and $y {\prec_C} x$, then $x=y$—entails that the causet cannot include causal loops. Since any event in such a loop precedes all the rest, these events would be considered the same event by the causet. Assuming that the emergent spacetime preserves the causal structure of the underlying causet, the emergent spacetime cannot contain closed timelike curves (or paths). While this assumption might, in principle, not hold, Wüthrich (2021, 469) concludes that “if causal set theory is regarded as a cosmological theory, there appears to be quite literally little space for an emergent spacetime to naturally accommodate closed timelike curves”. The theorem by Malament that served as the original motivation for CST (see Sect. 2.1) seem to further constrain which spacetimes can be approximated by CST: it shows that the causal structure of GR only determines the metric up to local volume information provided that spacetime is future- *and* past-distinguishing; if a causal set gave rise to a future- but not past-distinguishing spacetime (say), the causal structure of that spacetime would presumably not fix its metric up to the volume element—in clash with the Hauptvermutung’s idea of that causal sets lead to unique metric structures at lower energy scales.

That only a subset of GR models can, at best, be grounded in CST makes Baron and Le Bihan’s non-identity causal theory of spacetime vulnerable to one of the criticisms that they themselves refer to as that which defeated the identity causal theory of spacetime, namely that of Earman (1972). Earman’s criticism^9^ is exactly that a causal theory of spacetime cannot reproduce all GR spacetimes, including rather relevant ones. A causal theory of spacetime thus fails to get—in Earman’s words—“at the subtle and complex spatiotemporal relations which can obtain between events set in a relativistic space–time background” (Earman, 1972, 79). Baron and Le Bihan’s CST-based proposal has no new resources with which to address this worry. This is important to keep in mind when we, in Sect. 4, argue that a GR-based causal theory, which is otherwise effectively the one that Earman criticises, can do just as well as theirs.

## Grounding Spatiotemporal Relations

What Baron and Le Bihan want to ground in CST is “spatiotemporal relations”. By ‘spatiotemporal relations’ they mean aspects of GR, more precisely, those associated with the metric: “Core to the causal set theorist’s programme is the idea that the metric structure of spacetime [of GR] can be recovered from its causal structure” (Baron & Le Bihan, 2023, 2). Therefore, the most transparent statement of the aim of Baron and Le Bihan’s non-identity theory in the context of causal set theory is “to ground the metric connections in the ontology of causal set theory” (Baron & Le Bihan, 2023, 12).^10^

### Defining Spatiotemporal Relations

What, then, are these “metric connections” or, rather, relations associated with the general relativistic metric (where the metric itself together with a manifold comprise the spacetime as a whole)? Baron and Le Bihan mention a whole range of relations that, they propose, can be grounded in CST: “Spatiotemporal relations” (p. 3), “spatiotemporal distance” (p. 12), “timelike relations” (p. 12), “timelike separation” (p. 5), “timelike connections” (p. 11), “spacelike relations” (p. 5), “spacelike separation” (p. 5), “spacelike connections” (p. 10), “spacelike distance” (p. 12), $\cdots$.

Several of these are not really defined by Baron and Le Bihan, and getting clear on them is a first step towards understanding what the non-identity theory can, allegedly, do.

When stating their non-identity theory, Baron and Le Bihan seem to use ‘spatiotemporal relations’ as the collective term for all relevant spatiotemporal relations. It is, however, not immediately clear what Baron and Le Bihan might mean by ‘spatiotemporal distance’ in a general relativistic context. Consider the passage:

How are we to ground the metric connections in the ontology of causal set theory? Note that we are aiming to ground physical spatiotemporal distance relations represented in a more fundamental structure. In particular, what we are aiming to provide is a grounding story of the following broad kind: for spacetime points *x*, *y* and *z*, related via a spatiotemporal distance interval *d*, if $d(x, z) > d(x, y)$, then there are fundamental elements in the causal set structure (or groups of elements) *a*, *b*, *c* such that $d(a, b) > d(a, c)$. Thus, it is not the precise values for distance between points that we want to ground, but rather the relative distances between points (Baron & Le Bihan, 2023, 12)

The problem is that there is no notion of a spatiotemporal distance simpliciter on (*M*, *g*) in GR. One option towards such a simpliciter notion of spatiotemporal distance is to make recourse to what has been dubbed the null distance (see Allen & Burtscher, 2022; Sormani & Vega, 2016), but this requires a global time function as well (and is a somewhat experimental notion still under development in any case).

‘Timelike relations’ and ‘spacelike relations’ also seem to be meant as collective terms for those relations that specifically concern space and time, respectively. What in turn decides whether a relation between two events belongs to one or the other is whether the two events are timelike or spacelike separated. Timelike separated or, as they also say, timelike connected events are those where “one can be represented as located in the past or future light-cone of the other” (Baron & Le Bihan, 2023, 5). Spacelike separated or spacelike connected events are events that cannot be so located or, in other words, “spacelike separated events are ones that cannot be causally connected via a physical signal” (Baron & Le Bihan, 2023, 11). These physical signals are, as explained in Sect. 2.2, those that follow causal paths. ‘Timelike separated’ and ‘spacelike separated’ therefore differentiate between the events that are connectible via an everywhere timelike path, events related by $\prec_M$, and those which cannot. Notice for later purposes that there is always an everywhere spacelike curve between two points on a spacetime provided they are at all connected and standard topological properties granted.

The grounds for timelike and spacelike separation are then the following: The relation between “timelike separated events are grounded in the presence of causal relations between events [in the causet]. [...] Relations between spacelike separated events, by contrast, are grounded in the absence of causal relations between events [in the causet]. In particular, if two physical events are not linked by a fundamental causal relation, then that grounds a spacelike connection at the spatiotemporal level” (Baron & Le Bihan, 2023, 10). Following their understanding of causal relation explicated in Sect. 2.2, the fact that two events are timelike separated is grounded in the fact that there exists a causet chain between them that observes partial order, i.e., there exists a causal chain. The fact that two events are spacelike separated is grounded in the absence of such a causal chain.

The central aim for Baron and Le Bihan seems to be to provide a ground in causal set theory for “spacelike distance” and “timelike distance”. At least, causal set constructions for “spacelike distance” and “timelike distance” are what they discuss in most detail of the different spatiotemporal relations. However, they do not explicitly state what “spacelike distance” and “timelike distance” are, i.e, what they are seeking to ground. Simpliciter notions of timelike distance and spacelike distance are not primitive notions in GR nor are they frequently used. To find out what Baron and Le Bihan mean by “spacelike distance” and “timelike distance”, we here first give their grounding of these relations in causal set theory. From there, we attempt a charitable reverse engineering to the relations at the level of GR that they might be trying to ground like this while keeping in mind that the GR relations should qualify as the referent of “spacelike distance” and “timelike distance”. Only a minor change is needed to remedy their conception of “timelike distance” while “spacelike distance” remains problematic: we will offer an arguably more appropriate ground for “spacelike distance”, though its scope is ultimately limited.

### Timelike Distance

For reconstructing Baron and Le Bihan’s notion of “timelike distance”, we consider what Baron and Le Bihan propose as ground for this relation:

Timelike distances between two spacetime points are grounded in the number of causal links between two causal set elements. The distance between two spacetime points in the relativistic description thus corresponds to the shortest path through the causal set, between two causal set elements corresponding roughly to the two spacetime points. (Baron & Le Bihan, 2023, 12)

“Timelike distance”, in other words, is a relation that is proposed to be grounded in the length of a causal chain through the causet. More precisely, because there are typically many such causal chains between two events in the causet, they specify that “timelike distance” is grounded in the shortest chain as counted by the number of links needed to get from one to the other while observing the partial order. Because of the requirement that the embedding from the causet into the manifold must preserve causal structure, the number of links is a measure of the proper time of a corresponding timelike path^11^ in the GR model approximated by the causet. By “timelike distance”, Baron and Le Bihan therefore seem to refer to the proper time of a timelike path between the two events. Moreover, just as there is generally more than one chain between two events in the causet, there are more than one timelike path between two events. The proper time along each of these paths will generally differ; by identifying the ground for timelike distance with the shortest causal chain, they therefore seem to intend “timelike distance” to refer to the length of the timelike path between the two events with the least proper time. This, however, leads to a vanishing timelike distance value for *any* two timelike separated events in the limit and thus not at all to a fruitful notion of timelike distance.

Rather, we take them to have meant that timelike distance between two timelike-related events $x, y \in M$ is naturally defined as the proper time length of that path for which the proper time is *maximal* (or, rather, supremal), i.e.,

1 $$\begin{aligned} d_T(x, y):= \sup _{\gamma \in G(x, y)} \int _{\gamma } \sqrt{-\dot{\gamma ^a} \dot{\gamma ^b} g_{ab}} dt, \end{aligned}$$

where *G*(*x*, *y*) denotes the set of all timelike paths between *x*, *y*. This distance is also known as the Lorentzian distance. While many causal structures can be re-expressed and studied using the Lorentzian distance function (see Beem et al., 2017 for a comprehensive overview), it is important to realise that the Lorentzian distance function does not allow for turning the manifold into a metric space even if the Lorentzian distance is known between any two events; it thus cannot be used for studying convergence between metric spaces in analogy to how one does so with Riemannian distance, and in particular not serve as a complete substitute to the Lorentzian metric.^12^

Assuming the Lorentzian distance is what Baron and Le Bihan mean by timelike distance, we take them to have accordingly meant that the timelike distance is grounded in the (intrinsic) CST-timelike distance between two causal elements in a causal set which is standardly identified with the number of events on the *longest* causal chain connecting these two events.

This being said, the question arises as to whether they should only be interested in grounding a simpliciter notion of timelike distance between two events like this. There are, as mentioned, typically many timelike paths between two timelike separated events. We would rather argue that a causal theory of spacetime should ground the proper times of all timelike paths between two events since all of them qualify as the spatiotemporal relations between the two events, and not just their Lorentzian distance—especially because the resulting notion of Lorentzian distance is no sufficient substitute for metric structure to begin with. Baron and Le Bihan, however, do not seem to take the view that there are as many timelike relations as there are timelike paths between two events. Instead, their discussion proceeds as though there is one relation between two timelike separated events that needs grounding, their simpliciter notion of timelike distance.

### Spacelike Distance

Turning to a (putative) notion of “spacelike distance” between two events, things become more complicated. This notion is not explicated either by Baron and Le Bihan, but it seems safe to assume that two events are at a “spacelike distance” only if they are spacelike separated. Moreover, whatever the precise notion of “spacelike distance” between two events is to be, it is to be grounded in a causet despite the absence of a causal chain between the elements corresponding to the two sets; thus, spacelike separated events will have a chain between their corresponding elements in the causet, but this chain will involve going from preceding to succeeding events *and* from succeeding to preceding events, and will thus not observe the partial orders $\prec_C$ and $\prec_M$ (upon embedding).

Baron and Le Bihan offer the following CST ground for their notion of “spacelike distance”:

The distance between any two spacelike separated points is then grounded in the minimum number of relations of causal precedence it takes to arrive at a common causal ancestor within the underlying causal set description. In other words, the spacelike distance in the relativistic description is grounded in another notion of distance, expressed by the number of relations of causal precedence structuring the shortest path back to a common causal ancestor between two points. (Baron & Le Bihan, 2023, 12)

In accordance with the above, the ground for “spacelike distance” is the number of “relations of causal precedence” or, equivalently, the number of events in a causal chain. The chain is rendered as the shortest chain between one of the two events and a common ancestor, i.e., a third event from which there is a causal chain to both of the events whose spacelike distance is sought to be grounded. Since there are typically many common ancestors, one of them must be picked out like this, and they propose the one where the causal chain to the two events is shortest.^13^ Thus, their “spacelike distance” is grounded in a property of a chain that corresponds to a timelike path and the spacelike distance is given by the number of links in that chain.

Now, whatever “spacelike distance” is, grounding a simpliciter notion of spacelike distance in the least number of links back to a common ancestor seems inadequate. Here is why: The shortest causal chain to a common ancestor will correspond to the least proper time path to a common ancestor. In the GR model, that common ancestor will be at the intersection (if there is one) of the past lightcones of the two events. Consequently, the shortest time-like path to the common ancestor will be arbitrarily close to having proper time zero. While there may be no causal chain corresponding to exactly such a path, there must be one relatively close to it to satisfy the uniformity condition on the embedding. Thus, the shortest causal chain from a common ancestor to the events corresponds to a causal path with nearly vanishing proper time. Baron and Le Bihan’s ground for spacelike distance entails, in other words, that the distance between every two spacelike separated events approximately vanishes. But whatever “spacelike distance” refers to at the level of GR, such numerical degeneracy can hardly be appropriate.^14^

There is, however, a construction in CST that resembles that proposed by Baron and Le Bihan and which is sometimes referred to as a CST-counterpart to “spacelike distance” in GR (Rideout & Wallden, 2009, 5). This is also based on a common ancestor, but rather than looking at the chain from that ancestor to one of the events, the chain of interest is instead from a common ancestor to a common successor of the two events. Between every common ancestor and common successor, assuming both exist, there is at least one causal chain between them. The number of links in the longest of these chains gives the CST-timelike distance between them (as explained in the previous section). The spacelike distance, $D_s$, between the events, *x*, *y*, in the causet might then be proposed to be given by the shortest CST-timelike distance between a common ancestor, *w*, and a common successor, *z*, i.e,

2 $$\begin{aligned} D_s (x, y) = \text {min}_{(w, z) \in {\mathcal {I}}} D(w, z) \end{aligned}$$

where *D*(*w*, *z*) is the (intrinsic) CST-timelike distance, ${\mathcal {I}} = \{ (w, z) | w \in {\mathcal {J}}^+(x) \cap {\mathcal {J}}^+(y), z \in {\mathcal {J}}^{-}(x) \cap {\mathcal {J}}^{-}(y)\}$, and ${\mathcal {J}}^+(x):= \{y \in C | x \prec_C y \}$ (${\mathcal {J}}^-(x):= \{y \in C | y \prec_C x \}$) denote the causal future (past) of a causal set element. Rideout and Wallden (2009, 5) prefer to call this “naive spatial distance” because even also this construction is ultimately not satisfactory: *x*, *y* are always in the interval $[w_i, z_i]:= \{ u \in C | w_i \prec_C u \prec_C z_i\}$. Consequently, there is usually some *i* such that $[w_i, z_i] = \{x, y\}$ and so the shortest timelike distance is always 2. Various cures to the problem are discussed in Rideout and Wallden (2009).

Besides this, the question still remains what “spacelike distance” refers to at the level of GR. Here, Eq. (2) does come with the advantage that its counterpart, at least in Minkowski spacetime, measures the distance of a path that might reasonably be the bearer of a simpliciter notion of spacelike distance. The construction of Eq. (2) corresponds to an analogous construction at the level of GR as follows: consider two spacelike separated events *x*, *y* and the Lorentzian distance between a point *z* from the future cone intersection of *x* and *y* *and* a point *w* from the past cone intersection of *x* and *y* that is minimal, i.e. $d_S (x, y) = \text {inf}_{(w, z) \in I} d_T(w, z)$ where $d_T(w, z)$ is between $(w, z) \in I= \{ (w, z) | w \in J^+(x) \cup J^+(y), z \in J^{-}(x) \cup J^{-}(y)\}$, and $J^\pm (x)$ the usual notions of causal future/past. In Minkowski spacetime, a simple geometric argument entails that this Lorentzian distance is equal to the length of the unique spacelike geodesic linking *x* and *y*. For general spacetimes, however, this definition of “spacelike distance” simpliciter has problems. One central issue is that $d_S(x, y)$ is only well-defined if the set of pairs of past- and future-light cone intersection points *I* is non-empty. This, however, requires that the future cones of *x* and *y* intersect as well as their past cones which is not even guaranteed in globally hyperbolic spacetimes. (The future cones of two spacelike separated points in the interior region of Schwarzschild spacetime, i.e. inside the event horizon, may not intersect, for instance.) The problem is that there is still a spacelike path between *x* and *y*. Thus, by the proposed definition, *x* and *y* are still at a spacelike distance, but there is no way of measuring their spacelike distance, let alone in the corresponding causal set. One could qualify that events are only at a spacelike distance when $d_S(x, y)$ is well-defined, but this would entail that not all spacelike separated events are at a spacelike distance (see Sect. 3.1).^15^

Finally, it is worth pointing out that even if one is just interested in grounding the (now corrected) simpliciter notions of timelike and spacelike distances between spacetime points in the CST-spacelike and CST-timelike distances, it is still not as easily done as said. To see this, consider $\phi : {\mathcal {C}} \rightarrow [(M, g)]_{\rho _C}$, the faithful (and thus injective) embedding of the causal set in

$$\begin{aligned} {[}(M, g)]_{\rho {_C}}:= \{(M, g') | (M, g') \text { is isometric to } (M, g) \text { down to length scale } \rho _C \} \end{aligned}$$

which exists subject to the Hauptvermutung every time a causal set recovers a manifold spacetime (*M*, *g*). As said before, naively, one would want $d_T(x, y)$ (for *x*, *y* timelike separated) to be grounded in $D_T (\phi ^{-1}(x), \phi ^{-1}(y))$, and $d_S(x, y)$ (for *x*, *y* spacelike separated) to be grounded in $D_S (\phi ^{-1}(x), \phi ^{-1}(y))$. But even when fixating on one representative of the equivalence class—call it $(M, g')$—there is not a unique inverse: different sprinklings of points in $(M, g')$ are possible that give rise to different causal sets once these points are connected in agreement with the causal structure of (*M*, *g*); put slightly differently, the problem is that *not* every point of $(M, g')$ can be mapped onto *C* (*C* is of a lower cardinality after all); what needs to come in then is the fact there is a cut-off $\rho _C$—but how this comes in exactly (and what the sensible notion of a length cut-off can be to begin with) needs further technical scrutiny. It suffices here to say that the claimed grounding of timelike and spacelike relations in corresponding notions in CST—even when charitably reconstructed—is not a precise and thus arguably not a sure thing. This is worth keeping in mind for the reader when evaluating our proposal on how GR can in a robust sense be a causal theory of spacetime if CST was ever claimed to be one: the GR variant we propose will not be cursed by such an embedding issue (while the CST variant in the spirit of Baron and Le Bihan is cursed by it).

### Causal Chains as Grounds?

We have so far charitably reconstructed how Baron and Le Bihan see specific spatiotemporal relations grounded in causal set theory’s causal chains. In this section we now raise the question whether one could not—or rather even has to—ground spacetime structure, i.e., the metric field, as a whole. The straightforward advantage of such an approach seems to be that all derivative structures of the metric structure,^16^ structure which is itself clearly grounded in the metric, would be grounded as well by transitivity in the causet—this would include the simpliciter notions of spacelike and timelike relations that Baron and Le Bihan are explicitly after. More pragmatically speaking, one could then also latch onto how physicists recover the metric structure from the causet—see our discussion of how continuous spacetime approximates causets in Sect. 3—the grounding job would be done once the physicists are done recovering the metric structure.

Let us answer the central question of this section by making a small detour: we will wonder about which ground Baron and Le Bihan have precisely in mind when grounding their specific spatiotemporal relations of interest. This will lead us to find that not causal chains but really causets as such have to act as grounds in their non-identity theory of spacetime; but if so, then it will also follow that the metric can be seen as grounded, or so we will argue. (The causet as a whole is first and foremost the ground for the metric, whereby it seems unnecessary to take the further step to ground the individual relations.)

Now, Baron and Le Bihan themselves talk of the grounding base as “embedded in a total causal structure”, i.e. the causet, in their characterisation of the non-identity theory quoted in Sect. 2. Is this an admission that one is grounding in the causet? Or is the causet just meant as scaffolding structure within which the real, physical ground of causal chains happens to lie embedded for means of representation alone? The second option is plausibly what Baron and Le Bihan should have in mind if they are genuinely of the conviction that only causal chains in the causal set count as ‘causal’. Then, it would indeed make sense to have a strong aspiration that everything spatiotemporal needs to be grounded in causal chains as opposed to in the causal set structure as such. But if this is the underlying presumption, then it is in conflict with how the grounding project is actually carried out by them: as Baron and Le Bihan note themselves:

for [the grounding of spatial distance] to work the causal set structure must be a total causal structure, since only then will there always be a common ancestor in the structure for any two elements (this is just a reflection of the point noted above that there are no elements in a total causal structure that fail to be connected to at least one other element). (Baron & Le Bihan, 2023, 13)

As we see it, the causal chains in CST do not just physically exist qua causal chains alone—it is important that they weave together (in the right way) into a graph structure, or, employing a slightly different viewpoint, that they are embedded in a graph structure. An *isolated* chain does not correspond to a path as we know it from general relativity. Rather, the causet as a whole corresponds to a metric and manifold; and a causal chain can then be found to correspond to a specific path on that manifold by its place in the causet. Surely then, this destroys the prospect that spatiotemporal distance simpliciter can be grounded in a set of isolated causal chains alone (without any further structure imposed on that set).

Ultimately, then, we take Baron and Le Bihan’s reference to the total causal structure in this form as strong evidence that they themselves actually do not ground spatiotemporal relations *in causal chains alone* but really in the causal set. But then it is the question of why one does not ground the metric as a whole in the causet, which would then automatically allow for grounding the derivatives of the metric in the causet as well.

We can summarise the situation with a succinct dilemma for Baron and Le Bihan’s non-identity theory of spacetime:

1. If they consider spatiotemporal relations as grounded in causal chains alone, they need to provide a grounding strategy that can do without anything but causal chains, i.e., without the graph structure these chains are embedded in.
2. If they consider spatiotemporal relations as grounded in the whole causet, the whole metric (in line with the actual physical literature) can be grounded in the causet as well. Their whole construction is at risk of being redundant.

In fact, we take the problem to be worse than displayed by the dilemma alone. It is not just that we could also ground the metric as opposed to just some specific spatiotemporal relations in the causet. From all that what we know about GR today, it seems to us that we have to recover the metric structure as a whole (up to some approximation error). In any case, the conviction that one reconstructs general relativistic spacetime by simply sticking to some quasi-empirical or quasi-intuitive aspects of spacetime (such as a timelike or spacelike distances which supposedly do not depend on paths) is naive and has no backing in physical research.^17^ The advantage of taking this metric first approach to the causal theory of spacetime is also that it immediately avoids the problem of defining the relations that one is grounding (as detailed in Sect. 3.2 and 3.3) and arguing what relations require a ground. Once the metric is grounded, every well-defined relation derived from the metric will be grounded as well, nothing more, nothing less.

One might object that the causet as a whole is not causal and that this approach is therefore not true to the ambitions of a *causal* theory of spacetime. In reply, we would argue that the understanding of causation is already quite distorted in the approach where one is concerned with recovering specific spatiotemporal relations because of the need to appropriate it to the GR understanding of causation. Indeed, this understanding of causation seems to be nothing like that appealed to by, for instance, Smart (1969)—that Baron and Le Bihan also refer to—when he criticises the prospects for a causal theory of spacetime, though substantiating this claim will be postponed for future work.

## A Causal Theory of Spacetime from Relativity?

We have learned so far that Baron and Le Bihan seek to ground general-relativistically conceived simpliciter notions of timelike and spacelike relations in CST. Leaving aside that the precise grounding they had in mind was in need of correction (if it can work at all), and that one might want to ground timelike and spacelike relations relative to paths and not just simpliciter—most likely even the metric as a whole, we set out in this section to show that all that what Baron and Le Bihan do with their grounding of their simpliciter notions of timelike and spacelike relations in causal chains can equally be done within a causal re-construal of GR.

To reiterate, according to Baron and Le Bihan’s refined non-identity theory, “Spatiotemporal relations are grounded in the presence or absence of fundamental causal connections between events that are embedded in a total causal structure” (Baron & Le Bihan, 2023) where the total causal structure is given by the causet. We will now argue that exactly such a causal theory of spacetime can be given based on GR—we just need to take the total causal structure as given by GR rather than by CST. There might be problems with having such a causal theory of spacetime, but nowhere, or so we will argue in the following, are there problems which do not equally arise for Baron and Le Bihan’s causal set-based theory.

As mentioned already, the metric structure in GR is determined by a technical notion of causal structure, $(M,\prec_M)$, and the local volume element; the notion of causality is just the same causal-topological sense (which event—in the usual jargon—precedes which other event) as given by a causal set.^18^ A causal theory of spacetime within GR now takes the metric decomposition in this form as sufficient for an account of what spacetime is.

On the causal interpretation of GR, we will still make recourse to manifold structure. Let us explain why this is no problem in a fundamentally non-spatiotemporal but causal theory: the manifold on its own cannot sufficiently represent spatiotemporality. There is simply no structure for drawing the distinction between space and time. The manifold can thus be regarded as a mere scaffolding structure. As, for instance, Linnemann and Salimkhani (2021, 11) write:

First, it is essential to space and time (or spacetime) to play the role of an ordering structure. If the different concepts of time from physics to psychology and phenomenology have anything in common, it is the idea that time, among other things, is an ordering parameter. The same holds for space. Second, it is essential to space and time that the two are in a relevant sense distinct from one another. Given this view on the spatiotemporal, the manifold cannot count as spatiotemporal: *it is an ordering structure but lacks a distinction between one ordering parameter as opposed to the others.*

The idea of ‘local volume’ element which sounds at first spatial (thanks to ‘local’ and ‘volume’ in the label but, more severely, in the phrasing of how many events are in a ‘region’) is then really to be understood as an abstract region on that abstract scaffolding structure.

Now, the general relativistic timelike distance between two spacetime points can be grounded in the causal interpretation of GR as the number of events on the longest chain of events between the two points. Since GR works with a continuity assumption on the number of events, this number has to be infinite; to account for it, one makes use of the ‘local volume’ factor which determines the metric in addition to its causal topology. Secondly, the general relativistic spacelike distance $d_S(x, y) = \text {inf}_{(w, z) \in I} d(w, z)$ between *x*, *y* is grounded in the set of nulllike separated events emanating from *x* and *y* to *w* and *z* as well as those timelike separated events between *w* and *z*. This grounding in a causal interpretation of GR is possible for all future- and past distinguishing spacetimes, since the metric is here completely determined by all timelike paths (see Malament 1977). These are precisely the same spacetimes that one can, at most, hope to recover in CST, and the causal interpretation of GR can, therefore, at least ground the same spatiotemporal relations as the CST-based approach.

One might still wonder whether such a causal re-interpretation of GR is artificial in a way that CST is not. To answer this, let us in a first step rehearse the *general* motivations behind CST: the results of Hawking et al. (1976) and Malament (1977) ground the conviction that metric structure simply is (causal-topological) order and local volume; Rafael Sorkin, the originator of CST, put this reportedly as a slogan for CST as ‘number + order = geometry’. CST fleshes out this conviction explicitly with a discrete structure. Now, discreteness is hailed for various external reasons: discrete structures may seem less prone to contain representational artefacts than continuous structures, and discrete structures of gravity specifically may seem more in line with the goal of a quantum theory of gravity.^19^ More specifically to the context, however, discreteness may seem attractive for giving a way of obtaining the volume elements by counting events for free.

Baron and Le Bihan’s motivation for turning to CST is, however, another one: to provide a causal theory of spacetime. In this context, the decisive question is whether a discrete counting measure provides for a more genuine causal theory than a continuous measure *qua* causal theory. It is not clear at all that this should be the case. At most, one may say that a discrete causal theory is more parsimonious qua causal theory: for, one might want to argue that the causal theory from CST only requires a discrete causal graph structure, while the causal theory from GR assumes a continuous causal graph structure *plus* a manifold structure (Once framed in terms of measures, it is arguably more accurate to say that the counting measure is a simpler measure than any continuous measure). It strikes us though as if this advantage can only be cashed out at a superficial level, neglecting the difference between expressing a discrete and continuous set of events: naturally, when you talk of a continuous structure you need different technical apparatus, but is it thereby necessarily less parsimonious?

On the other hand, as stressed already towards the end of Sect. 3, it is at this stage technically unclear how the more ambitious aim of grounding spacelike and timelike relations in causal chains (rather than in the causal set as a whole) goes through—an issue which the causal theory of GR does not have to wrestle with in this form: the grounding of $d_S(x, y)$ for $x, y \in (M, g)$ spacelike related requires well-defined counterparts for *x*, *y* on the causal set but not all elements of *M* have a counterpart in the causet that grounds (*M*, *g*). Again, maybe the grounding relation can be rescued but more work needs to be done in spelling out the precise embedding of *x*, *y* here. (Even if only *x*, *y* are to be grounded that are part of length-cut-off manifold, as arguably the picture of Baron and Le Bihan, how is this exactly to work?)

It is a bit beside our main point of this section to ask why one should commit to such a causal re-interpretation of GR vis-à-vis other interpretations of GR, since the aim was rather to establish that the non-identity theory à la Baron and Le Bihan need not make recourse to CST. For completion, let us nevertheless close this section by considering the viability of the causal interpretation of GR. Firstly, one could ask what motivates a re-interpretation of GR that prefers the inner of the light-cone to one that makes use of the outer of the light-cone.^20^ In other words, could one not also have proposed a non-identity theory of spacetime based on the spacelike relation? Well, the known results about recovering metrical properties from topological properties are all based on the inner-light cone structure (Hawking et al., 1976; Malament, 1977). In fact, spacelike connectibility seems simply not sufficiently differentiated as to ground a topological reconstruction: “In higher [than 2] dimensional spacetimes there is always a spacelike curve from any point to any other, but not always a timelike one. What is crucial here seems to be that time (but not space) is one dimensional” (Nerlich, 1982, 386). Secondly, a heads-on objection against the causal re-reading is that it unnecessarily throws away the natural resources of spacetime *geometry*. But note that we are not deflationary about spacelike distances simpliciter on a causal interpretation of GR—we are just re-reading them in different lights by giving the values attached to them a different understanding (namely, in terms of a number of events).

## Conclusion

We have charitably reconstructed Baron and Le Bihan’s grounding of spatiotemporal relations and thereby their non-identity theory of spacetime. However, we found the resulting non-identity theory to be still lacking in a couple of ways: While the reconstruction salvaged their idea of grounding a notion of timelike distance, the grounding of a notion of spacelike distance was challenged both by what exactly this distance is in relativity, to begin with, and whether the proposed ground was even defined for a large enough class of spacetimes. Next, we argued that their specific grounding project is, at least, as successful in the theory of GR as it is in CST. The latter holds no new resources for realising a causal theory of spacetime. Finally, we argued that the grounding story is much more plausibly run via the metric field by which the explicit grounding of the specific derivative structure relative to the metric, however, obtains only an illustrative purpose. All work is done, once the causet is found that (approximately) corresponds to the metric and manifold of interest.

## Footnotes

1 Modulo the fact that GR can still be interpreted in a spatiotemporal fashion, unlike CST.

2 We use the word ‘events’ for both causal set elements and points in the spacetime manifold. Which notion is meant, should become clear from the context.

3 Notably, one talks of both the causet approximating the continuum spacetime and the continuum spacetime approximating the causet; technically, they will be linked to maps which are inverses of one another, see below.

4 A spacetime (*M*, *g*) is called future-distinguishing if two points $p, q \in M$ which share the same chronological future are the same, i.e. $I^+(p)=I^+(q) \implies p = q$. The chronological future $I^+(p)$ is hereby defined as $I^+(p):= \{ q \in M | p \ll q\}$ where for $x, y \in M: x \ll y$ if there exists a future-directed timelike path from *x* to *y*; the causal future is defined as $J^+(p) = \{ q \in M | p \prec _M q\}$; analogous definitions hold for past-distinguishing, chronological past and causal past. Any further definitions can be obtained from any standard reference on general relativity.

5 For gentle conceptual introductions to CST, see for instance Sorkin (2006), Dowker (2013) and Surya (2019) and the introductory sections of Wüthrich and Callender (2017, 2); we will in fact follow the nomenclature of the latter.

6 We will mainly follow Surya (2019) for this.

7 See, however, Saravani and Aslanbeigi (2014) for a discussion of other options.

8 But cf. Nerlich (1982) for an influential dissenting view.

9 For a critical reconstruction of Earman’s criticism, see Wüthrich and Huggett (2020).

10 ‘Connection’ in ‘metric connections’ is to be charitably understood as standing for the metric structure more generally and not for the technical sense of connection in differential geometry.

11 The proper time for a path $\gamma : [a, b] \subset {\mathbb {R}} \rightarrow M, t \mapsto \gamma (t)$ is defined as $\tau := \int _a^b dt \sqrt{-g_{\mu \nu } {\dot{\gamma }}^{\mu } {\dot{\gamma }}^{\nu }}$.

12 Nevertheless, the Lorentzian distance is a useful concept for many spacetimes: for all globally hyperbolic spacetimes (*M*, *g*), the Lorentzian distance function is finite and continuous on $M \times M$; note also that, for all strongly causal spacetimes, this notion is a natural generalisation of the local distance function, which is available on any Lorentzian manifold. See Beem et al. (2017, Ch 4).

13 An ambiguity is lurking here, since the number of events on the chain from the common ancestor to one of the two events might not be the same as the number of events on the chain from the common ancestor to the other event. (Does one for instance use the larger or the smaller of the two distances to a fixed common ancestor when comparing the distances between the pair and their different common ancestors?) We will set this issue aside since we will ultimately argue that this construction does not work in any case.

14 A sensible notion of spacelike distance between *x* and *y* in terms of timelike paths should in general agree with the notion of length of the shortest possible spacelike geodesic linking *x* and *y* (or rather that of the infimum of the lengths of all possible spacelike geodesics between the two points).

15 One may note that a notion of spatial distance in terms of the infimum of lengths of connecting spacelike geodesics is, at least at times, available. However, this intrinsically spacelike notion of spacelike distance does not offer itself for an immediate grounding into a corresponding causal set theory structure: there are no counterparts to spacelike geodesics in the causal set.

16 Examples include: the projective structure, the conformal structure, the spatial and temporal arclength of spacelike and timelike paths respectively, etc.

17 Cf. the failure of constructive axiomatic approaches in *reducing* general relativistic spacetime structure. What they do is help explicate it in terms of empirically flavoured notions of particle and light trajectories. See Linnemann and Read (2021), Adlam et al. (forthcoming).

18 We discussed earlier what problems are linked to associating causality with this prima facie technical notion of causal connectedness of relativity (and also causal sets). But these are problems which concern the causal theory from CST just as they concern the proposal here from GR.

19 Cf. for instance Wüthrich (2023, 4): the main motivation, I take it, is that the fundamental structure which will give rise to relativistic spacetimes is assumed to be a discrete structure because this is what one could expect, perhaps on the basis that in quantum theories, many physical observables have discrete spectra.

20 See Nerlich (1982, section 5) for criticism of such a re-interpretation with focus on the inner light-cone in the context of SR.
